# Is There a Need for Vitamin D Supplements During Summer Time in Northern Germany? A Study of Hospitalised Fracture Patients

**DOI:** 10.3390/nu16234174

**Published:** 2024-11-30

**Authors:** Steffi S. I. Falk, Guido Schröder, Thomas Mittlmeier

**Affiliations:** Clinic of Trauma, Hand and Reconstructive Surgery, University of Rostock, Schillingallee 35, 18057 Rostock, Germany; guido.schroeder@med.uni-rostock.de (G.S.); thomas.mittlmeier@med.uni-rostock.de (T.M.)

**Keywords:** osteoporosis, osteoporosis-associated fractures, vitamin D, nutrient supplements, bone metabolism

## Abstract

Background/Objectives: Vitamin D is a key factor in bone metabolism, especially in patients who have suffered fractures, a group in need of a healthy bone metabolism. In Germany, a 70-year-old person requires 20 min of sun exposure daily for sufficient endogenous production in April. While this appears to be a sufficient period on paper, it raises the question of whether sufficient synthesis is achieved, given the time and the implementation of skin cancer prevention. Furthermore, it is necessary to determine whether self-medication is a safe option. Methods: This was an analysis of vitamin D levels in patients with fractures over a one-year period. To avoid bias due to vitamin D intake, patients were divided into groups (self-medication, without, prescribed). The differences due to age, gender, fracture type and fluctuation over the year were analysed. Results: 613 patients with a mean age of 73 years (45–97) were enrolled. The mean vitamin D level across all groups was 51 nmol/L, with a mean of 40 nmol/L for patients without supplementation (*n* = 449). Monthly comparisons revealed significant differences between January/February and August/September. Similarly, a comparison by gender showed a significant difference (*p* = 0.028). However, there were no significant differences between osteoporosis-associated and non-osteoporosis-associated fractures. Conclusions: The majority of patients did not achieve sufficient vitamin D levels through endogenous synthesis and substitution did not lead to toxic levels. This suggests that substitution is reasonable and safe even during the summer months.

## 1. Introduction

1,25-Dihydroxyvitamin D (1,25(OH)2D3) plays a significant role in bone metabolism. It promotes bone mineralization by regulating calcium and phosphate metabolism. Sufficient vitamin D is therefore essential for maintaining healthy bones, regardless of whether osteoporosis exists. The results of recently published meta-analyses have confirmed the existence of a correlation between low serum vitamin D levels and an elevated risk of fracture [1,2]. Vitamin D differs from other vitamins in that the body can synthesise it from endogenous precursor substances. The synthesis of vitamin D occurs endogenously and is dependent on the exposure of the skin to ultraviolet B (UVB) radiation from the sun.

Additionally, dietary sources of vitamin D are available. It is important to distinguish between plant-based vitamin D2 and animal-based vitamin D3. Vitamin D2 has been demonstrated to exert a markedly inferior effect on the elevation of serum vitamin D (25(OH)D3) levels in comparison to vitamin D3. An adequate intake of vitamin D3 through dietary sources could be achieved, for instance, through the consumption of 100 g of wild salmon, 2 L of full-fat milk, or 10 eggs per day. However, findings from studies conducted in Germany indicate that only 10 to 20 percent of the vitamin D3 requirement is met through food sources [3].

Vitamin D deficiency in infancy and childhood results in the insufficient mineralisation of the bones, leading to soft and deformable bones (rickets). In adults, a lack of vitamin D leads to a disturbance in bone metabolism. The result can be a lack in bone mineralisation (osteomalacia) which can contribute to the development of osteoporosis. Additionally, deficiency is discussed as a potential cause of pseudarthrosis [4].

In order to prevent vitamin D deficiency, a vitamin D3 supplement is given to children in Germany for the first 1.5 years of life. Beyond that period, guidelines recommend only adequate sun exposure to promote endogenous vitamin D3 synthesis for the rest of life. A review of the existing literature indicates that individuals over the age of 18 require 800–1000 IU, or 20 μg, of vitamin D3 daily [5,6,7].

In order to ensure an adequate daily endogenous production of vitamin D3 in northern Germany outside of the winter months, an average of 20 min of face and arm exposure to the midday sun daily is estimated to be sufficient in March. The required time decreases to six minutes in June and July and to 12 min in September [8]. These results are calculated on the basis of the synthetic capacity of the skin of a young adult aged 20 years. With advancing age, the capacity of the skin to synthesise vitamin D3 declines. A 40-year-old individual will retain a capacity of 75%, necessitating a duration of 26 to 16 min. The synthesis capacity of an elderly person of 70 years old is 50% that of a young adult of 20 years. Accordingly, the required time interval is twice as long [9]. It should be noted that the aforementioned times are contingent upon exposure to ultraviolet radiation on the exposed surface of the skin, namely the face and arms, in the absence of any sun protection measures.

In northern Germany, the human body is incapable of synthesising sufficient vitamin D3 to meet daily requirements between the months of October and March. This is due to the reduction in UVB radiation, which results in lower levels of sunlight. In order to ensure an adequate supply of vitamin D, it is essential to replenish the body’s reserves during the summer season. The level of solar exposure in northern Germany is comparable to that experienced in other major cities, including Berlin, London, York, Moscow, Minsk, Calgary and Brussels.

The question thus arises as to whether sufficient endogenous synthesis is achieved, given the limited synthesis time and the implementation of skin cancer prevention. Furthermore, it is necessary to determine whether self-medication with vitamin D3 is a safe option for patients. The focus of this study was on patients with fractures, a group in definite need of a healthy bone metabolism.

## 2. Materials and Methods

The data presented here were obtained from a single-centre study carried out at a university hospital of Rostock. Patients were enrolled over a period of twelve months starting in April 2021 in the department of trauma surgery.

In this study, only patients aged 45 years and older were consecutively included. The inclusion criteria were as follows: the patient must have sustained a fracture after low-energy trauma (for example, a stumbling fall) which resulted in hospital admission. Patients with underlying tumorous disease and chronic high-grade renal failure were excluded from the study. All patients provided informed consent for the collection and analysis of their data.

Vitamin D (25(OH)D3) levels were quantified in blood samples obtained from all patients at the University of Rostock laboratory. The Institute for Clinical Chemistry and Laboratory Medicine is accredited as a laboratory in line with the standards defined by DIN EN ISO 15189 and DIN EN ISO 22870. The vitamin D serum value (25(OH)D3) was determined using the ELICA ligand assay. To avoid any bias in the data due to the use of vitamin D3, patients were interviewed about this and then divided into three groups. The first group included patients with vitamin D3 supplementation, the second group included patients without supplementation, and the third group included patients with a prescribed intake. Blood vitamin D (25(OH)D3) levels were classified according to the recommendations of the German Society for Nutritional Medicine, as shown in Table 1.

In addition, the patients without vitamin D3 intake were divided into groups according to the type of fracture they had. This grouping process made it possible to analyse the vitamin D (25(OH)D3) levels for the most common types of fracture. In particular, it allowed for comparisons between major osteoporotic (major osteoporotic fractures are distal radial fractures, proximal humerus fractures, spine fractures and proximal femur fractures) and non-osteoporotic-associated fractures similarly to how comparisons would be made between genders.

Furthermore, the minimal time for sufficient endogenous daily vitamin D3 production was calculated based on the geographical degree of longitude and latitude of the University hospital of Rostock (N54.0924, E12.0991; 18 m elevation at sea). The calculations were made for the endogenous synthesis of 800 IU on the 14th of each month, based on the following assumptions: The body is exposed to sunlight to the extent of 25%. Individuals with skin type 2 were the most common skin type group in the patient population. The evaluation considers three age groups and the current sunburn threshold without skin protection [8].

The analysis of the descriptive data was carried out using Microsoft Excel 2019. SPSS version 28 was used for further statistical analysis of the data. The Kolmogorov–Smirnov test was utilised to analyse the data for normal distribution, and it identified a deviation from normal distribution. For pairwise comparison of two groups, the Mann–Whitney U test was utilised, whereas for assessing more than two groups, the Kruskal–Wallis test was used. Given the considerable number of tests required for statistical analysis, Bonferroni correction was employed to ensure the reliability of the results. *p* values lower than alpha were considered significant (alpha = 0.05).

## 3. Results

A total of 613 participants with an average age of 73 years were included in the study, of whom 447 were women. The demographic characteristics of the study population are presented in Table 2. A statistically significant difference was identified between the first two groups and the last group (prescribed intake) in terms of vitamin D (25(OH)D3) levels (*p* = 0.0001). Nevertheless, no significant difference in age was observed between the cohort of patients who did not take vitamin D3 and those who took vitamin D3 in the form of dietary supplements (*p* = 0.688).

**Figure 1:**
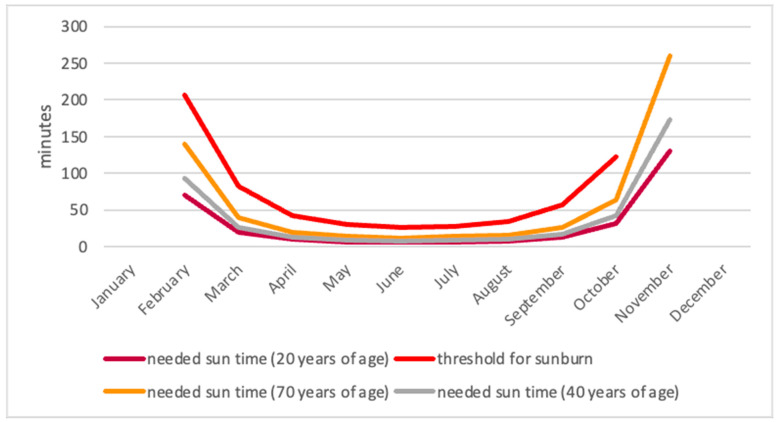
The amount of time required for the synthesis of endogenous vitamin D3 to reach a sufficient level in unprotected skin at different ages. The calculations were made for the endogenous synthesis of 800 IU on the 14th of each month, based on the following assumptions: latitude is 54.0924° and longitude is 12°09′91″. The elevation at sea level is 18 m. The body is exposed to sunlight to the extent of 25%. Individuals had skin type 2. From November to February, there is an insufficient amount of endogenous synthesis from a mathematical standpoint. The calculated time for the onset of sunburn is indicated by red shading [8].

Figure 1 below illustrates the minimum level of UVB radiation (sun exposure) required by individuals in different age groups for sufficient endogenous vitamin D synthesis during the year. Additionally, the figure provides the maximum duration of unprotected sun exposure that is deemed to be safe, and for which sunburn is highly unlikely to occur. It can be observed that all age groups have the capacity for sufficient endogenous synthesis of vitamin D at midday from March to October.

**Figure 2:**
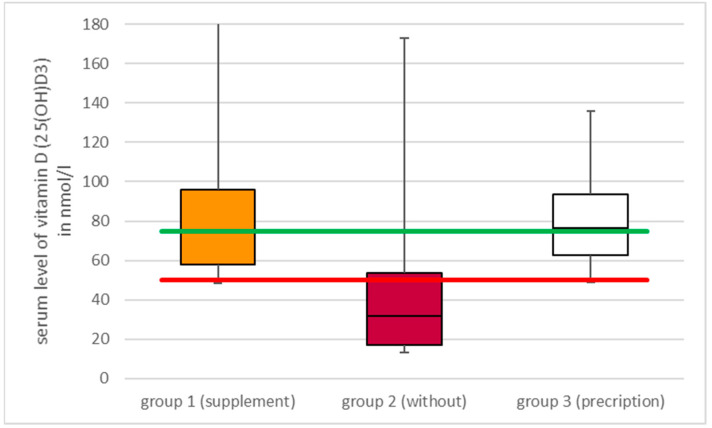
The box plots presented here illustrate the serum vitamin D (25(OH)D3) levels in the aforementioned three groups, classified according to their respective vitamin D3 intake. Additionally, the red line represents the lower limit value for a vitamin D (25(OH)D3) undersupply, while the green line indicates the lower limit value for an adequate vitamin D (25(OH)D3) supply in the serum.

The serum vitamin D (25(OH)D3) values were determined. Considering all patients in all groups, the mean level of vitamin D (25(OH)D3) was 55 nmol/L. The group with a dietary supplementation of vitamin D3 achieved a result of 82 nmol/L. In contrast, the group that did not receive any supplementation exhibited the lowest mean value with an average of 40 nmol/L (Figure 2). The group that received prescribed vitamin D3 supplementation exhibited an average value of 76 nmol/L. A significant difference between the group without vitamin D3 supplementation and the other two groups was revealed by the statistical analysis (*p* = 0.0001), while no significant difference was observed between the groups with vitamin D3 supplementation (*p* = 0.719). Therefore, substitution results in elevated serum levels of vitamin D (25(OH)D3), irrespective of whether the decision to do so is made by the patient or recommended by the physician.

**Figure 3:**
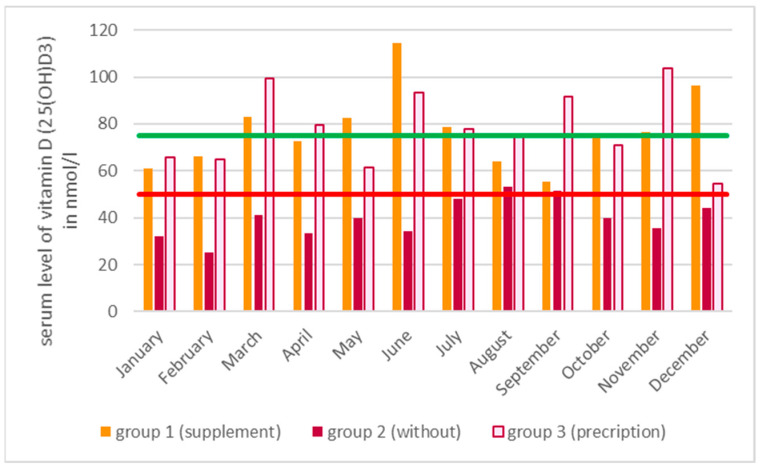
The distribution of vitamin D (25(OH)D3) serum values throughout the year is illustrated, with values separated according to the intake of vitamin D3. Additionally, the red line represents the lower limit value for a vitamin D (25(OH)D3) undersupply, while the green line indicates the lower limit value for an adequate vitamin D (25(OH)D3) supply in the serum.

Across all groups, the mean level of vitamin D (25(OH)D3) showed fluctuations throughout the year, as illustrated in Figure 3. The statistical analysis revealed no statistically significant differences in the mean age of patients for each individual month (*p* = 0.657). Additionally, no significant differences were observed in the vitamin D (25(OH)D3) levels between months within group 1 (with supplement) (*p* = 0.504) or group 3 (with prescription of Vitamin D3) (*p* = 0.066). The Bonferroni-corrected month-by-month comparisons for group 2 (without supplementation) revealed statistically significant differences between January and August (*p* = 0.025), February and August (*p* = 0.001), and February and September (*p* = 0.042).

**Figure 4:**
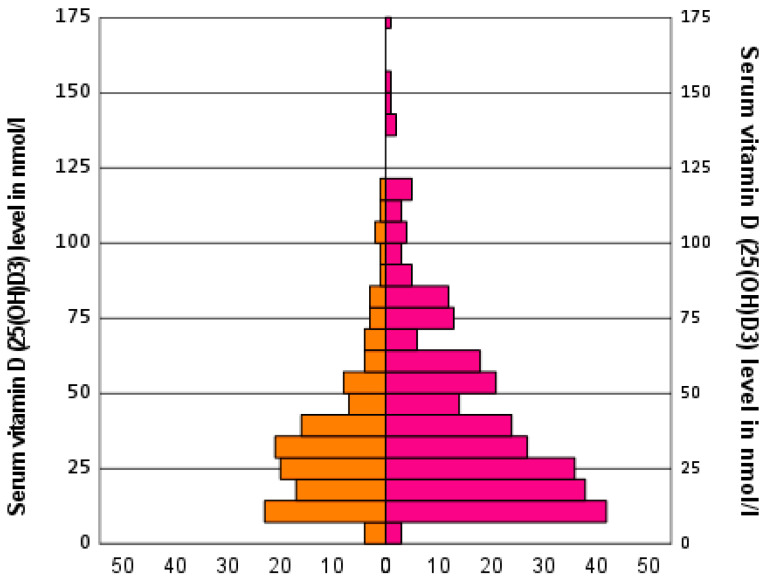
Serum concentration of vitamin D (25(OH)D3) as well as its distribution by gender in patients who did not receive any supplementary vitamin D3. The horizontal axis represents the number of patients. The male patients are represented on the left side of the diagram and the female patients are represented on the right side.

The mean value of serum vitamin D (25(OH)D3) exhibited fluctuations within a range of ±3 nmol/L when the two age groups were subdivided into those aged between 45 and 69 years and those aged 70 years and above. This analysis was conducted on the basis of the three defined groups of vitamin D3 intake. The younger age group tended to have lower values, as seen in Table 3. Among the patients who did not take vitamin D3 (group 2), there was no significant difference between the two age groups (*p* = 0.316). A comparison of the serum vitamin D (25(OH)D3) levels by gender (Figure 4) in this group revealed a statistically significant difference (*p* = 0.028) between males (34 nmol/L) and females (43 nmol/L).

One patient reached a critical level of vitamin D (25(OH)D3), with a level of 485 nmol/L, respectively, without any clinical symptoms. She was a 73-year-old patient with a self-determined vitamin D3 dietary supplementation.

**Figure 5:**
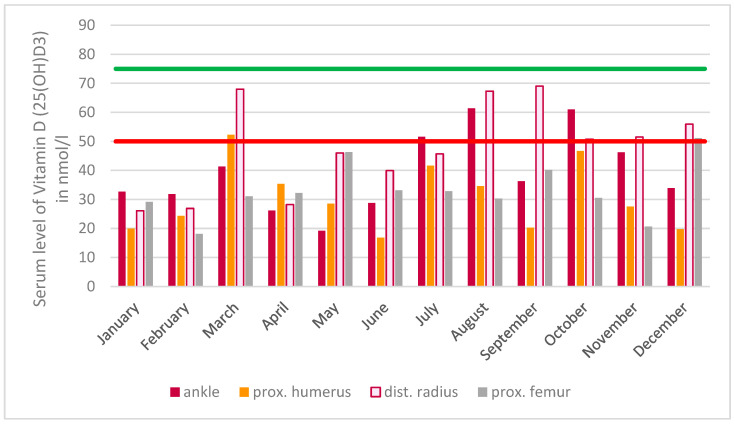
A comparison of serum vitamin D (25(OH)D3) levels over a one-year period in patients who did not receive vitamin D3 supplementation (group 2) was conducted on two patient cohorts: those with ankle fractures (as distinct from fractures associated with osteoporosis) and those with major osteoporotic fractures. Additionally, the red line represents the lower limit value for a vitamin D (25(OH)D3) undersupply, while the green line indicates the lower limit value for an adequate vitamin D (25(OH)D3) supply in the serum.

The distributions and vitamin D (25(OH)D3) values shown in Figure 5 were obtained for the subgroup of patients without a vitamin D3 intake (group 2) and are presented according to the fracture types of the long bones (proximal humerus, distal radius, proximal femur and ankle fractures). This analysis considers the classical traumatic fractures, only. Even when the data were divided into two groups, one comprising major osteoporotic fractures and the other comprising non-osteoporosis-associated fractures, no significant differences in vitamin D (25(OH)D3) serum levels were observed (*p* = 0.630).

## 4. Discussion

The aim of this study was to obtain an overview of the vitamin D (25(OH)D3) status among patients with fractures treated surgically, as healthy bone metabolism is a prerequisite for bone healing. The questions addressed were whether sufficient synthesis of vitamin D3 is possible in consideration of the prevention of skin cancer and whether this occurs in reality. In addition, there was a question to be answered as to whether any vitamin D3 supplementation taken by patients is safe or leads to increased hypervitaminosis. The data collected demonstrate that despite sufficient UVB radiation, sufficient endogenous synthesis of vitamin D3 was not achieved. Without an adequate supply, it is also impossible to replenish the body’s own stores for the winter months, resulting in a vitamin D deficiency for the entire year. Moreover, the data also show that substitution, whether self-administered or prescribed, is a safe procedure. The proven serum vitamin D (25(OH)D3) deficiency was also independent of age and the fracture suffered.

The calculation of the required sun exposure and the sunburn threshold, taking into account the decreasing synthesis capacity with advancing age, indicated that sufficient endogenous vitamin D3 synthesis is feasible until old age from March to October. The required synthesis time is below the sunburn threshold for all calculated ages. Consequently, the human body is able to synthesise an adequate amount of vitamin D3 in our latitudes from March to October with sufficient UVB irradiation.

In accordance with the inclusion criteria, this study includes a patient cohort with advanced age. This is evidenced by the mean age of 73 years. Despite the inclusion of patients aged 45 and above, the population under study can be considered to be predominantly geriatric. This is to be expected in view of the increasing incidence of fractures with age and is also reflected by the number of patients in the over 69 age group. In terms of gender distribution, there was a disproportionately large number of women compared to the overall population. However, this observation is relativised when taking into account that these were exclusively fracture patients. Given that women have a lifetime fracture risk of 40% and this corresponded to 13% for men, a gender distribution of 4:1 is representative for the group of fracture patients [11].

A period of 12 months allows for a comprehensive overview of the annual course in the target group of fracture patients, as demonstrated by the inclusion of 613 patients. This provides a reliable statement for the annual course in the analysed target group of patients with fractures. In addition, the examination of additional living conditions, such as housing situation and outdoor activity, enables a better understanding of the characteristics of the cohort of fracture patients and reveals a homogeneous distribution of characteristics between the groups defined. The only noteworthy finding is that a greater proportion of patients in the group with prescribed vitamin D3 intake lived in a nursing home. This finding is also consistent with the results of Wabe et al. [12], who were able to show that 68% of nursing home residents were prescribed vitamin D3.

A notable strength of this study was the consideration of the potential influence of vitamin D3 supplements on the observed results. The authors’ research indicates that this information is missing from the results and that the inclusion and exclusion criteria were unclear in the majority of published studies on vitamin D serum levels [13]. Consequently, this represents a limitation of their study results. The laboratory values obtained, namely 173 nmol/L for the group without supplementation and 9 nmol/L for the group with supplementation, indicate that the data derived from medical history may not be entirely reliable. However, this was not to be expected for anamnestic data. Nevertheless, the fraction of 27% of patients with a medical history of vitamin D3 supplementation shows that this proportion was so large that it should be considered in the analysis.

According to the mean value across all groups of 51 nmol/L, the target value for a possibly sufficient supply of vitamin D (25(OH)D3) of 51 nmol/L would be just reached (Table 1) [10]. For the group without vitamin D3 intake, it was shown that the patients were not sufficiently supplied with vitamin D during the summer months, despite the possibility of endogenous synthesis, regardless of the patient’s age. If the safe supply limit of 75 nmol/L is taken as the lower limit value, it becomes evident that this was not even reached in a single month of the study period. It is also noteworthy that even with supplementation, despite an annual mean value above 75 nmol/L, this lower limit value, which is considered safe, was not reached in all months (Figure 3). Thus, even with substitution, there were no reliably sufficient serum levels found over the entire year. This endemic vitamin D deficiency situation is confirmed in the literature via studies of other segments of the population [13,14,15,16].

The analysis of the different age groups did not reveal significant differences and thus corresponded to the findings of Gannage-Yared et al. [17] among hospital employees. The study by Lee et al. [14] had demonstrated that young women had significantly lower serum levels. Conversely, Jang et al. [18] showed that older patients with distal radius fractures tend to have lower vitamin D (25(OH)D3) serum levels. Therefore, further clarification is required.

The seasonal fluctuations with the lowest values in February and the highest in August were also reported by Bleizgys and Kurovskij [19] and Cabral et al. [20]. This is consistent with the expected difference, given that sufficient vitamin D3 synthesis is no longer possible from October onwards. Consequently, the body must then rely on stored reserves until endogenous synthesis can be initiated again in March/April.

A comparison of serum vitamin D (25(OH)D3) values between the sexes revealed a significantly lower value for men. A limited number of studies have previously described this comparison, with divergent results. In contrast to our study, Yan et al. [15] showed the opposite outcome in a randomly selected cohort of 300 adults. It is important to note that the mean age of their participants was 46 years, which is significantly lower than that of the patients studied here. Moreover, their patients did not have any fractures. The authors posit that the primary reason for this discrepancy was the age of the patients. In contrast, Gannagé-Yared et al. [17] did not observe any gender-specific differences in serum vitamin D (25(OH)D3) levels among 392 hospital employees. The cohort in their study was also notably younger, with an average age of 42 years. Subramanian et al. [21] were able to investigate the associations between sociodemographic characteristics and vitamin D (25(OH)D3) levels using representative data for the United States. Their findings indicated that male gender is a risk factor for vitamin D deficiency. In addition, their data showed that vitamin D deficiency in women occurred especially in the age group of reproductive women, which only forms a very small group in this study.

In accordance with the study of Bleizgys and Kurovskij [19], the current study did not demonstrate any age-specific difference in serum vitamin D (25(OH)D3) levels. On the other hand, Diaz et al. [22] were able to prove in their study that the risk of vitamin D deficiency was significantly higher for women than for men. However, it should be noted that this study was carried out among employees of an university in Ecuador with an average age of 41 years. Consequently, the female participants were at an age at high risk of vitamin D deficiency, which could explain their difference compared to the values presented here. Similarly, Yan et al. [15] found a higher prevalence of vitamin D deficiency in women and at the same time showed that there was a significant difference between young and older women. Given an average age of 42 years, it is not feasible to make a comparison with the patient group presented here. It can be assumed that the average serum values for vitamin D (25(OH)D3) would be different if the high-risk group (young women) were excluded.

The condition of hypervitaminosis was observed in only one female patient. In their study, Bleizgys and Kurovskij [19] observed the occurrence of hypervitaminosis, particularly in children, a group that was not included in the present study. A review of the literature by the authors revealed a lack of analyses examining the incidence of hypervitaminosis in individuals undergoing vitamin D3 supplementation.

The administration of vitamin D3 supplementation resulted in a statistically significant elevation in serum vitamin D (25(OH)D3) levels when compared to the non-supplemented group. This was observed irrespective of whether patients ingested vitamin D3 independently or if it were prescribed it by a medical practitioner. The authors of the study believe that the results in the group taking a nutritional supplement (group 1) demonstrate an increasing recognition of the importance of vitamin D in the general population. Concurrently, however, the data also indicate that the patients analysed here lack familiarity with dose determination. This is also reflected in the considerable standard deviations observed in the groups with Vitamin D3 intake (group 1 and 3), resp. It is not possible to conclude from our data if patients in that groups had received initial counselling from their general practitioner. Given the minimal toxic levels attained through supplementation, a routine intake in both winter and summer seems reasonable and safe—even if only the patient monitored the intake regime. This can therefore be considered in terms of osteomalacia prophylaxis in fracture patients. From the authors’ perspective, the data suggest that such counselling or improved advice on vitamin D3 supplements would be beneficial. Further studies are needed to clarify the degree to which these results can be transferred to the general population including younger patients.

### Limitations

It must be acknowledged that the data are limited by the exclusion of patients under the age of 45 and the focus on patients with fractures. Additionally, some patients were unable to provide a clear description of the contents of their supplements, which may have resulted in some degree of misclassification. This unknowledgeable intake of vitamin D3 would result in an increase in the mean values observed in the group that did not receive supplementation. As the data already indicate an undersupply, any incorrect allocation in this group has no significant negative effects. A further limitation is the fact that the subgroups were sometimes too small to allow for an analysis. However, by calculating the minimum group size for an analysis, it was possible to prevent underpowering. Despite this limitation, the group of patients without vitamin D3 substitution was of sufficient size to allow for a subgroup analysis to be carried out in this group with the most widespread intake behaviour. With regard to the research question, this was the most important group.

The application of minimal sun exposure enabled the assessment of the viability of endogenous synthesis without the occurrence of sunburn. Given the many factors affecting synthesis performance, it was vital to identify the specific skin type under consideration [23]. The predominant skin type observed among the study participants was designated as 2, which was used as the basis for defining the skin type for the study. It must be acknowledged that the results are not transferable to other skin types or geographical regions. It should be noted however that this definition reflects the collective of patients under consideration.

A further limitation of this study is that not all participants were able to provide specific information regarding their vitamin D doses. Furthermore, the large number of products from different manufacturers also contributed to this limitation. With regard to the patients who provided information, preparations containing 400, 1000, and 2000 IU were identified in the group of patients who were taking dietary supplements. The majority of patients indicated that they were taking 1000 IU. In the domain of prescribed vitamin D supplements, patients were predominantly administered 20,000 IU every 14 days, with occasional instances of 1000 IU daily. Nevertheless, this reflects the reality and therefore the question of whether vitamin D supplementation can be placed in the hands of our patients—in other words, whether it is safe—can be answered without this information. Furthermore, it could be demonstrated that the group with supplementation is so large that this information should be taken into account in further studies.

## 5. Conclusions

It can be concluded that neither the summer nor the winter requirement of vitamin D is covered by endogenous synthesis in the older population. Calculated sun exposure data for the months of March to October indicates that it is feasible to meet the body’s vitamin D3 requirement through synthesis. However, data collected for northern Germany suggests that target serum levels are not achieved even during the summer months, despite the replenishment of vitamin D stores for the winter months. The majority of patients did not achieve sufficient vitamin D (25(OH)D3) levels through endogenous synthesis. Furthermore, in one case, substitution led to toxic levels. Therefore, it can be reasonably and safely assumed that substitution is appropriate and safe even during the summer months. This should be considered with regard to osteomalacia prophylaxis in fracture patients. Additionally, patients undergoing supplementation should be provided with more detailed information to enhance their understanding of the intake regimen and facilitate more effective intake. The extent to which these results can be extrapolated to the normal population needs to be clarified in further studies.

## Figures and Tables

**Table 1:** Definition of vitamin D (25(OH)D3) status based on serum vitamin levels [3,10].

Values in nmol/L	Values in ng/mL	Category
<12	<4.80	serious deficiency
12–30	4.80–12	deficiency
30–50	12–20	insufficient intake
50–75	20–30	probable insufficient intake
75–100	30–40	sufficient optimal
100–150	40–60	probably too high
150–375	60–150	hypervitaminosis
>375	>150	toxic level

**Table 2:** Details on the demographics based on the vitamin D3 intake of the study participants.

	Group 1 (Supplement)	Group 2 (Without)	Group 3 (Prescription)	Over All Groups
patients	96	449	68	613
Age (years)	73 (46–93)	72 (45–97)	81 (48–97)	73 (45–97)
Gender				
Male	16 (17%)	147 (33%)	3 (4%)	166 (27%)
Female	80 (83%)	302 (67%)	65 (96%)	447 (73%)
BMI (kg/m^2^)	26 ± 6	27 ± 6	25 ± 6	26 ± 6
Living environment				
Living independently	83 (86%)	363 (81%)	44 (65%)	490 (80%)
Home with assistance	5 (5%)	47 (10%)	10 (15%)	62 (10%)
Nursing home	8 (8%)	39 (9%)	14 (20%)	61 (10%)
Walking aids				
None	63 (66%)	306 (68%)	30 (44%)	399 (65%)
Walking stick	7 (7%)	29 (7%)	6 (9%)	42 (7%)
Crutches	7 (7%)	20 (4%)	1 (1.5%)	28 (5%)
Rollator	15 (16%)	80 (18%)	28 (41%)	123 (20%)
Wheelchair	2 (2%)	12 (3%)	1 (1.5%)	15 (2.5%)
Immobile	1 (1%)	2 (0,5%)	2 (3%)	5 (1%)
Leaving home				
Daily	76 (79%)	322 (72%)	36 (53%)	434 (71%)
Weekly	12 (13%)	88 (20%)	20 (29%)	120 (20%)
Monthly	4 (4%)	15 (3%)	2 (3%)	21 (3%)
Never	4 (4%)	22 (5%)	10 (15%)	36 (6%)
Fracture mechanism				
Bicycle	8 (8%)	50 (11%)	2 (3%)	60 (10%)
Ground-level fall	77 (80%)	346 (77%)	62 (91%)	485 (79%)
Fall over a last step	10 (10%)	28 (6%)	4 (6%)	42 (7%)
Other	1 (1%)	25 (6%)	0	26 (4%)
Nutrition				
Mixed diet	90 (94%)	444 (99%)	67 (98,5%)	601 (98%)
Vegetarian	6 (6%)	5 (1%)	1 (1.5%)	12 (82%)
Vegan	0	0	0	0

The age is given in years mean (min-max), the BMI is given as mean ± standard deviation, and all other numerical values are presented as a counted number with an associated percentage.

**Table 3:** Demographic details of the groups and serum vitamin D (25(OH)D3) levels in the subgroup analysis according to age.

	Group 1 (Supplement)	Group 2 (Without)	Group 3 (Prescription)	Over All Groups
45–69 years				
Patients	39	195	11	245
Male	3	86	1	90
Female	36	109	10	155
Age (years)	61 (46–69)	60 (45–69)	63 (48–69)	60 (45–69)
Vitamin D (nmol/L)	82 ± 41 (9–205)	40 ± 27 (4–153)	73 ± 27 (19–114)	48 ± 34 (4–205)
69 years and older				
Patients	57	254	57	368
Male	13	61	2	76
Female	44	193	55	292
Age (years)	81 (70–93)	82 (70–97)	84 (72–97)	73 (45–97)
Vitamin D (nmol/L)	81 ± 61 (22–485)	40 ± 30 (5–173)	77 ± 41 (14–136)	52 ± 41 (5–485)
Vitamin D (nmol/L) over all ages	82 ± 54 (9–485)	40 ± 29 (4–173)	76 ± 25 (14–136)	51 ± 38 (4–485)

The values are presented as numbers, and for the serum levels of vitamin D (25(OH)D3), as mean ± standard deviation (minimum to maximum).

## Data Availability

The data presented in this study are available on request from the corresponding author due to privacy and ethical restrictions.
